# Development and Feasibility Testing of PROMPT-Care, an eHealth System for Collection and Use of Patient-Reported Outcome Measures for Personalized Treatment and Care: A Study Protocol

**DOI:** 10.2196/resprot.6459

**Published:** 2016-11-24

**Authors:** Afaf Girgis, Geoff P Delaney, Anthony Arnold, Alexis Andrew Miller, Janelle V Levesque, Nasreen Kaadan, Martin G Carolan, Nicole Cook, Kenneth Masters, Thomas T Tran, Tiffany Sandell, Ivana Durcinoska, Martha Gerges, Sandra Avery, Weng Ng, Stephen Della-Fiorentina, Haryana M Dhillon, Ashley Maher

**Affiliations:** ^1^ Centre for Oncology Education and Research Translation Ingham Institute for Applied Medical Research Liverpool Australia; ^2^ Department of Medicine South Western Sydney Clinical School University of New South Wales Sydney Australia; ^3^ Liverpool Cancer Therapy Centre Liverpool Hospital Liverpool Australia; ^4^ Illawarra Cancer Care Centre Wollongong Hospital Wollongong Australia; ^5^ Centre for Oncology Informatics University of Wollongong Wollongong Australia; ^6^ Illawarra Health and Medical Research Institute University of Wollongong Wollongong Australia; ^7^ Cancer Institute New South Wales Sydney Australia; ^8^ Macarthur Cancer Therapy Centre Campbelltown Hospital Campbelltown Australia; ^9^ Faculty of Science Central Clinical School The University of Sydney Sydney Australia; ^10^ Didymo Designs Wollongong Australia

**Keywords:** patient-reported outcomes, eHealth, self-management, real-time report, oncology, patient-centered care, PROMPT-Care, electronic medical records, oncology information systems

## Abstract

**Background:**

Patient-reported outcome (PRO) measures have been used widely to screen for depression, anxiety, and symptoms in cancer patients. Computer-based applications that collect patients’ responses and transfer them to the treating health professional in real time have the potential to improve patient well-being and cancer outcomes.

**Objective:**

This study will test the feasibility and acceptability of a newly developed eHealth system which facilitates PRO data capture from cancer patients, data linkage and retrieval to support clinical decisions and patient self-management, and data retrieval to support ongoing evaluation and innovative research.

**Methods:**

The eHealth system is being developed in consultation with 3 overarching content-specific expert advisory groups convened for this project: the clinical advisory group, technical advisory group, and evaluation advisory group. The following work has already been completed during this phase of the study: the Patient-Reported Outcome Measures for Personalized Treatment and Care (PROMPT-Care) eHealth system was developed, patient-reported outcomes were selected (distress, symptoms, unmet needs), algorithms to inform intervention thresholds for clinical and self-management were determined, clinician PRO feedback summary and longitudinal reports were designed, and patient self-management resources were collated. PROsaiq, a custom information technology system, will transfer PRO data in real time into the hospital-based oncology information system to support clinical decision making. The PROMPT-Care system feasibility and acceptability will be assessed through patients completing PROMPT-Care assessments, participating in face-to-face cognitive interviews, and completing evaluation surveys and telephone interviews and oncology staff participating in telephone interviews.

**Results:**

Over the course of 3 months, the system will be pilot-tested with up to 50 patients receiving treatment or follow-up care and 6 oncology staff at 2 hospitals in New South Wales, Australia. Data will be collected to determine the accuracy and completeness of data transfer procedures, extent of missing data from participants’ assessments, acceptability of the eHealth system and usefulness of the self-management resources (via patient evaluation surveys and interviews), and acceptability and perceived usefulness of real-time PRO reporting (via oncology staff interviews) at the completion of the pilot phase.

**Conclusions:**

This research investigates implementation of evidence into real world clinical practice through development of an efficient and user-friendly eHealth system. This study of feasibility and acceptability of the newly developed eHealth system will inform the next stage of larger scale testing and future implementation of the system as part of routine care.

**ClinicalTrial:**

Australian New Zealand Clinical Trials Registry ACTRN1261500135294; https://www.anzctr.org.au/Trial/Registration/TrialReview.aspx?id=369299&isReview=true (Archived by WebCite at http://www.webcitation.org/6lzylG5A0)

## Introduction

Patient reported outcome (PRO) measures have been widely used in a variety of settings including screening for depression, anxiety, and symptoms in cancer patients [1,2] and in the primary care [3] and rural settings [4]. There are many examples of computer-based applications that collect patients’ responses and translate them in real time into a useable format for the treating health professional [1,2,5-8]. A systematic review undertaken by Chen et al [5] concluded that routinely collecting PRO measures enables better patient-centered care in cancer settings when a patient management plan is integrated with routine collection of PROs. This review identified strong evidence that well-implemented electronic PRO (ePRO) systems with timely feedback improved patient–health care provider communication and patient satisfaction and may also improve the monitoring of treatment response and detection of unrecognized problems [5]. The impact of ePROs on clinical and health service outcomes has also now been demonstrated with a large randomized controlled trial with cancer patients reporting significant outcomes including reduced emergency room visits, longer tolerability of chemotherapy, and improved survival [9].

We have previously published a discussion paper citing a lack of ePRO systems being implemented as part of routine oncology care in Australia and detailing our intention to develop an eHealth system, Patient-Reported Outcome Measures for Personalized Treatment and Care (PROMPT-Care), which has the potential to lead to improvements in patients’ quality of life and health outcomes while reducing variations in cancer outcomes [10]. This PROMPT-Care system is based on a prototype system, PROsaiq, which was previously developed to import PRO surveys into a hospital’s Oncology Information System (OIS) [11]. This protocol paper reports on the steps involved in developing this clinical system and the proposed testing of its acceptability and feasibility in 2 oncology care centers. At the time of publication, some of the work described was completed and some was pending.

The purpose of the PROMPT-Care eHealth system is to support the routine collection and analysis of PROs from cancer patients over time, from the time of being registered as a cancer patient, and make this information available to the patient and the health professionals involved in the delivery of their cancer care. The eHealth system will also deliver evidence-based self-management information to address patient-reported problems and empower patients to take a more active role in decision making and managing their ongoing care and recovery. Importantly, one of the key features distinguishing PROMPT-Care from previous eHealth systems used in the oncology setting is its integration into the hospital’s point-of-care OIS.

The term “patient” used throughout this document encompasses all people diagnosed with cancer during and after their acute treatment phase including into longer term survivorship.

## Methods

### Study Design and Objectives

This is a feasibility study with the overall aim of developing and testing an integrated eHealth system to support and enable cancer patients to achieve and maintain improved health and well-being and better cancer outcomes.

The specific objectives are as follows:

1. Develop an eHealth system that is integrated into the hospital’s OIS (MOSAIQ, Elekta Medical Systems, Sunnyvale, CA) to support assessment of cancer patients’ PROs through the use of electronically administered standardized assessment tools, provision of real-time feedback of the results to the treating clinicians, and generation of links to self-management resources for patients that are tailored to their PROs. This includes developing a production version of the PROsaiq prototype system [11].

2. Implement a pilot version of PROMPT-Care at 2 hospitals and test the feasibility and functionality of the system.

3. Test the acceptability of the pilot version of PROMPT-Care in a sample of cancer patients and clinicians at the 2 participating hospitals.

### Setting

The feasibility study will be undertaken in the cancer centers of 2 public hospitals in New South Wales (NSW), Australia. Liverpool Hospital is the largest of the 6 hospitals in South Western Sydney Local Health District, a district with a population of over 820,000 people, comprising 12% of NSW residents. The communities in this district are socially, economically, culturally, and linguistically diverse, and the area contributes 10% of the total new cases of cancer load in NSW. Liverpool Hospital treats more than 81,000 patients annually. Wollongong Hospital is the largest of the Illawarra Shoalhaven Local Health District’s 9 hospitals. This district has a population of more than 390,000 residents. and Wollongong Hospital, the region’s tertiary referral hospital, treats more than 47,000 patients annually.

### Ethics Approval and Consent to Participate

Ethics approval was obtained from the Human Research Ethics Committee of South Western Sydney Local Health District with site-specific ethics approvals obtained for Liverpool Hospital and Wollongong Hospital.

### Development of the PROMPT-Care eHealth System

The PROMPT-Care system includes key features relating to system design, data collection, assessment reporting, and workflow integration that were identified from a review of 33 ePRO systems as being important to supporting a successful ePRO system [1]. We have previously summarized these recommended features [10].

#### Establishing Clinical, Technical and Evaluation Advisory Groups

A total of 3 expert groups were convened to inform the development and evaluation of the PROMPT-Care system: a clinical advisory group (CAG) and a technical advisory group will guide the content, development, and functionality of the system including transformation of PROsaiq from prototype to production, and an evaluation advisory group will guide the feasibility and acceptability testing of the developed system. Additionally, special working groups were convened, as required, to advise on specific aspects of the eHealth system development and content. The membership and roles and responsibilities of these groups are presented in Table 1.

**Table 1:** Patient-Reported Outcome Measures for Personalized Treatment and Care (PROMPT-Care) project advisory group membership, roles, and responsibilities.

Membership and expertise		Roles and responsibilities	Special working groups
**Clinical advisory group**			
	Core members (n=38) with a range of expertise: radiation and medical oncology, nursing, allied health, psycho-oncology, hospital management, cancer systems innovation management, OIS^a^ and electronic records management, and research.	Responsible for the overall content and implementation of PROMPT-Care^b^ as part of routine care, including the clinician feedback system and the patient self-management system. The CAG^c^ is responsible for decisions about: -The outcomes of cancer patients to be collected over time -The PRO^d^ measures to collect the agreed patient outcomes -The frequency of measurement of the PROs -The modes of delivery of the PRO information to health professionals involved in the delivery of patient care in both summary and longitudinal format -The PRO score thresholds which will trigger recommended actions by the clinical team -The content of the evidence-based recommendations to be generated in response to each PRO that is above the predetermined threshold -The suite of evidence-based self-management information that will address patient-reported problems and enable patients to take an active role in decision making and managing their ongoing care and recovery	A clinical algorithms working group will be specifically focused on development of algorithms and evidence-based recommendations for clinicians, which are required for programming the clinician feedback reports. A self-management working group will identify suitable self-management resources for patients using the PROMPT-Care system.
**Technical advisory group**			
	Core members (n=23) with expertise in cancer systems innovation, OIS and electronic records management, hospital information management and technical design, oncology informatics, and medical and radiation oncology.	Responsible for overseeing the development and implementation of the production information technology system (PROsaiq) and infrastructure to support PRO data capture and management including: -Integration of the pilot PROMPT-Care system with the existing hospital information technology systems and OIS to support real-time data access to all members of the care team, including network configuration, with special consideration of hospital security firewalls -Delivery of a patient assessment interface suitable for use on both desktop and mobile technology (eg, tablets) to support PRO data capture either within the clinic or from home at predefined periods	A MOSAIQ reporting working group will be specifically focused on display of the PRO data in MOSAIQ.
**Evaluation advisory group**			
	Core members (n=10), with wide-ranging research and statistical expertise, particularly in psycho-oncology and clinical research.	Responsible for developing an evaluation plan to document the feasibility and acceptability of the PROMPT-Care system.	

^a^OIS: oncology information system.

^b^PROMPT-Care: Patient-Reported Outcome Measures for Personalized Treatment and Care.

^c^CAG: clincial advisory group.

^d^PRO: patient-reported outcome.

#### Selection of Patient-Reported Outcome Measures and Assessment Frequency

The CAG was consulted about which PRO domains were most important for informing patient care and amenable to evidence-based intervention. The CAG was presented with the following domains to consider: symptoms, distress, anxiety, depression, quality of life, and unmet needs. Following the selection of the domains to be assessed, a comprehensive review of specific measures was undertaken to select the final core set of PRO measures. PRO measures that met the following recommended properties were favored for inclusion in the PROMPT-Care survey: simple, brief, informed by patients, reliable, valid and responsive to change, easily scored and interpreted, and free to use as well as those which predicate clinical action [12]. In an effort to minimize patient burden, an item map was developed during this phase to identify any significant duplication of items across the short-listed measures, and any redundant measures were excluded from the core PRO assessment. The final measures selected were: the Distress Thermometer (DT) [13] with the problem checklist [14], the Edmonton Symptom Assessment Scale (ESAS) [15] and the Supportive Care Needs Survey–Screening Tool 9 (SCNS-ST9) [16]. However, it is noteworthy that once the system has been set up and tested, changes in the PRO measures can be made in the future (ie, the initial decisions regarding PROs are not locked in long-term).

The CAG was also consulted about the frequency of patients completing the PRO assessments, with consideration given to (1) the timeframe for the response options for each of the selected PRO measures (eg, within the past week), (2) allowing sufficient time between 2 assessments for clinical recommendations to have been actioned, (3) minimizing patient burden and therefore improving compliance, and (4) whether the assessment frequency should differ for patients on-treatment versus those in follow-up. The CAG advised on the frequency of assessments for this phase of the PROMPT-Care program while acknowledging that the feasibility and acceptability testing would inform future assessment frequency.

#### Development of Algorithms to Guide Response to Patient-Reported Outcomes

For each of the selected PRO measures, item and scale cut-off scores differentiating between normal (below threshold) and clinical (above threshold) responses were determined from published sources [14,16,17]. These threshold scores informed the development of clinical and self-management recommendations. A multidisciplinary clinical algorithms working group was convened (medical and radiation oncologists, social worker, clinical psychologist, care coordinators) to develop actionable recommendations for each item that breaches the clinical threshold. A total of 15 actionable recommendations were developed after consultation with published guidelines [14,18]. These recommendations were tailored to the specific issue of concern (eg, symptom vs information need), and they ranged from “No action required” to “Clinically address as appropriate OR refer to [*types of specialties indicated here, depending on issue*] for further assessment and care.”

#### Development of Patient-Reported Outcome Feedback Reports

The PROMPT-Care system is designed to allow any oncology staff member from the participating cancer centers to access their patients’ PRO assessment reports. A total of 2 report formats were developed in consultation with the CAG members: (1) a summary report of the patient’s most recent PROMPT-Care survey, which included recommendations for the care team to address the patient-reported concerns and (2) a longitudinal report summarizing the PROs over time to allow the clinical team to identify trends and determine whether previously implemented interventions have addressed patients’ issues of concern. Consideration was given to the content and presentation of the feedback reports with a focus on minimizing any need for interpretation of scores and highlighting issues of concern (scores above predetermined thresholds) in red to readily draw attention to them. Refer to Multimedia Appendices 1 and 2 for examples of the clinician summary and longitudinal feedback reports.

#### Collation and Review of Patient Self-Management Resources

The PROMPT-Care assessment measures items in the domains of physical well-being (eg, fatigue, pain, mouth sores), emotional well-being (eg, anxiety, depression, loss of interest in activities), social and family well-being (eg, support from family and friends, problems with partner), and practical support (eg, transport, housing, being informed about test results). A self-management working group was established as a subgroup of the CAG to identify suitable, readily available self-management resources in each of these domains as well as in the “maintaining health and well-being” domain for general health issues. Identified resources were systematically reviewed on the basis of their quality (language used, links active and relevant, peer-reviewed resource, HonCode certification [19], currency, applicability and objectivity). Each resource was reviewed by a member of the working group, and the results were collated on an evaluation form that outlined whether or not the resource was to be included in the pilot project. Resources were sought from local NSW cancer websites and organizations in the first instance followed by reputable Australian sources and finally from international cancer organizations. These self-management resources will be accessible to participating patients via 5 domain-specific pages hosted on the Cancer Institute NSW (CINSW) eviQ website [20], with patients’ responses to the PROMPT-Care assessment determining which pages they were able to access.

### Participants

As this is a feasibility study, staff will be selected on the basis of their willingness to comprehensively test the PROMPT-Care system and provide feedback to inform any modifications required for the next phase of research. Clinicians will be asked to identify eligible patients for the pilot study who they perceive would also be willing to provide comprehensive feedback on the pilot PROMPT-Care system. Hence, the participant selection was purposive rather than representative.

Eligible patients are people who are either currently receiving cancer care (including follow-up care) or have recently been diagnosed with cancer and are scheduled to commence cancer treatment at one of the participating sites. Eligibility criteria include a confirmed diagnosis of cancer, age 18 years or over, cognitively able to provide informed consent and understand the surveys, and sufficient skills to complete the survey in English. Exclusion criteria are having a diagnosis of a blood cancer and not having access to the Internet outside of the clinic.

All staff who provide care in the oncology departments at the participating hospitals are eligible to participate. However, as this is a small feasibility study, 3 clinicians from each hospital (6 in total) who were not directly involved in the development of any aspects of the PROMPT-Care system will be invited to participate. In this phase of research, only 3 staff were ineligible to participate due to their direct, rather than advisory, role in building the technical or clinical components of the PROMPT-Care system.

### Measures

The system’s functionality will be assessed over a 3-month period with some patients expected to complete only 1 PROMPT-Care assessment during this period (if recruited later in the study) and some completing up to 3 assessments (if recruited at the start of the study).

- The accuracy and completeness of data transfer procedures (from the point of the patient completing an assessment to a report appearing via MOSAIQ) will be assessed by comparison of data received by MOSAIQ to data presented in the clinical feedback reports.
- The extent of missing data from participants’ assessments will be assessed through examination of the PROMPT-Care reports to determine whether there is any systematically missing data.

In this study, the main purpose of assessing acceptability is to identify any modifications necessary for improving patient and provider uptake of the system in the next phase of research. Hence, acceptability of the system to patients and staff will be assessed as follows:

- Patients will complete an evaluation survey and interview at the end of the pilot phase to determine their perceptions of acceptability of the eHealth system and usefulness of the self-management resources.
- Cancer center staff directly involved in the pilot phase will participate in an evaluation interview at pilot study completion. The interview will focus on their perceptions of the acceptability and perceived usefulness of the real-time PRO reporting.

### Procedure

#### Oncology Team Training

During the set-up phase, oncologists and other staff (including nurse care coordinators and allied health staff) from the 2 participating cancer centers will be introduced to the PROMPT-Care program through presentations made by the chief investigator and directors of cancer services at both sites. They will receive training resources which include background information about the purpose of PROMPT-Care, the battery of PROs and interpretation of their outcomes, information on how to access the summary and longitudinal reports via their OIS, and strategies for discussing the PROMPT-Care outcomes with the patient. One-on-one and group meetings will be held with all clinicians involved in this feasibility phase to facilitate familiarity with and high utility of the PROMPT-Care system.

#### Patient Recruitment

Given the small number of patients required for the feasibility study, participating clinicians will review their patient lists for the upcoming 4 to 6 weeks to identify patients who meet the eligibility criteria. Research staff will then mail an information and consent pack to eligible patients and will telephone patients 2 weeks after mail-out to confirm receipt of study materials, answer any questions about participation, and initially obtain verbal consent. Patients who require a replacement invitation pack will be sent another one immediately. Patients who return a signed consent form or who provide verbal consent when phoned by research staff will be asked to attend a PROMPT-Care appointment 20 minutes prior to their upcoming scheduled appointment at the cancer center in order to complete study paperwork (including written consent if not already received) and their first PROMPT-Care assessment. Patients who are unable to be reached before their next scheduled clinic appointment or who require more time to consider their participation will have the opportunity to consent at a later time and complete a PROMPT-Care assessment prior to another upcoming clinic appointment. Research staff will be available to assist patients who need help completing the surveys.

#### PROMPT-Care Assessments

An assessment schedule will be established when the participant enters the study that indicates the frequency and pattern of assessments that the participant will receive. Patients who are on-treatment will complete the PROMPT-Care survey every 2 to 4 weeks, depending on the schedule of their review appointments. Patients on follow-up will complete assessment approximately monthly. It was agreed that patients completing the PROMPT-Care survey every 2 to 3 weeks would complete only the DT/Checklist and ESAS on every occasion with the SCNS-ST9 added to the battery for every second assessment, as that measure has been validated using a 4-week time frame. Participants completing the PROMPT-Care survey on a monthly basis would always complete the full assessment (ie, DT/Checklist, ESAS, and the SCNS-ST9).

Patients who are attending the clinic will complete the PROMPT-Care survey in the waiting area using an electronic tablet device provided by the research team. Follow-up patients will typically complete their PROMPT-Care survey from home via a link sent by email. However, if follow-up patients are attending the clinic for a review appointment, they will complete their monthly survey while in the waiting area. As this is a pilot project to determine the feasibility and acceptability of the PROMPT-Care system, patients who are due to complete their PROMPT-Care surveys from home will be sent a reminder email if they have not completed it within the requested timeframe (48 hours).

#### Access and Review of Reports

To facilitate rapid access and review of the patients’ PRO reports, all patients participating in this PROMPT-Care feasibility study will be flagged as “PROMPT-Care Trial” participants on the OIS used by the participating sites. Clinicians are instructed to access the report during the consultation, review any issues flagged as problematic by the patient (ie, scores above threshold), discuss these with the patient, and take any appropriate actions to address the issues.

#### Patient Self-Management

Upon completion of the PROMPT-Care assessment, patients will receive an email with links to the website page(s) for each domain in which they breached threshold scores on any of the items in that domain. For example, if any of the physical domain items were breached, the link to that page would be included in the patient’s email; if not, that link would be excluded. Patients who scored below threshold on all items would only receive the link to a “maintaining health and well-being” page.

#### Evaluation of Acceptability of PROMPT-Care

The purpose of the acceptability assessment is to identify any modifications required to the PROMPT-Care system in preparation for phase 2 of our research. Patients and oncology staff will participate in the assessment of system acceptability.

Cognitive interviews are a technique that will be used to assess patient understanding of the survey questions and response options. This technique requires participants to verbalize their thoughts as they process and answer questions in an attempt to identify issues pertaining to comprehension, inability to retrieve relevant data to accurately answer questions, errors in wording, and whether there is a discrepancy between the lived experience and response options in the survey [21]. The PROMPT-Care study will use a combined think-aloud and verbal probing technique [22] with standardized verbal prompts at various parts of the survey while also allowing spontaneous probes based on participant observations (eg, I noticed you started several thoughts when considering that answer. Can you tell me a bit more about them?) and conditional probes (eg, I noticed you paused for a long time before you answered. Could you tell me why?). A subset of participants will volunteer to take part in the cognitive interviews, which will be conducted during the first time patients complete the PROMPT-Care measures. The cognitive interviews will be recorded using Camtasia (TechSmith Corp) software and are expected to take approximately 45 minutes.

Participating patients will be asked to complete an evaluation survey and invited to participate in a brief telephone interview to determine their views on the eHealth system and the usefulness of the self-management resources at study completion.

Oncology hospital staff who have had direct contact with PROMPT-Care feasibility study patients will be invited to participate in a brief semistructured telephone interview to assess their views on the eHealth system at study completion. The interview will focus on determining staff’s perceptions of technical issues relating to ease and timeliness of accessing the PROMPT-Care reports, usefulness of the reports’ content and format, perceived impact (positive or negative) on workload, and perceived need for training to support wider scale implementation of the PROMPT-Care system.

## Results

Descriptive data will be collected to inform accuracy and completeness of data transfer from the PROMPT-Care surveys to the OIS. All interviews with patients and oncology staff will be audiorecorded and transcribed verbatim with quotes extracted indicating patient and staff reflections on their engagement with the PROMPT-Care system and identification of access barriers or problems. Cognitive interviews will be reviewed and patient cognitive errors identified to highlight potentially problematic elements of the survey.

## Discussion

This research investigates implementation of evidence into real world clinical practice through development of an efficient and user-friendly eHealth system to facilitate (1) PRO data capture, (2) data linkage and retrieval to support clinical decisions and patient self-management, and (3) data retrieval to support ongoing evaluation and innovative research. The system includes PROs which have been identified by a clinical advisory group as being appropriate and relevant for the clinical setting, overcoming documented barriers of acceptability and relevance [5]. Integration of the PRO measures into the existing hospitals’ OISs enhances their relevance and usefulness in informing routine cancer care.

The data collected will inform the feasibility and acceptability of this system-level strategy and identify barriers which should be addressed to facilitate wider implementation of this system in clinical practice. Once fully established, the accumulated data from the PROMPT-Care system will inform population-level needs of cancer survivors to identify potential gaps in care. The systematic approach to data collection over time will also allow the assessment of the impact of changes in service delivery over time. In the future, the system can be adapted to collect PROs from the non-English–speaking cancer community, thereby extending our understanding of the needs of this vulnerable and underresearched group.

## Appendix

Multimedia Appendix 1 Clinician summary feedback report.

Multimedia Appendix 2 Clinician longitudinal feedback report.

## Abbreviations

CAG: clinical advisory group
CINSW: Cancer Institute New South Wales
DT: Distress Thermometer
ePRO: electronic patient-reported outcome
ESAS: Edmonton Symptom Assessment Scale
NSW: New South Wales
OIS: Oncology Information System
PRO: patient-reported outcome
PROMPT-Care: Patient-Reported Outcome Measures for Personalized Treatment and Care
SCNS-ST9: Supportive Care Needs Survey–Screening Tool 9 items
