# Supplementary material for: Development and Feasibility Testing of PROMPT-Care, an eHealth System for Collection and Use of Patient-Reported Outcome Measures for Personalized Treatment and Care: A Study Protocol
Source: JMIR Res Protoc. 2016 Nov 24;5(4):e227. doi: 10.2196/resprot.6459 (PMC5146324; doi:10.2196/resprot.6459)
Supplement: Multimedia Appendix 1 [file resprot_v5i4e227_app1.pdf]

**** MOSAIQ eCERT LCTC *****

PROMPT Care
Patient Reported Outcome Measures for Personalised Treatment and Care

Patient Name: ZZZ, VICKY	MRN: 12345678	DOB: 27/06/2010	Date of Assessment: 11/12/2014

THIS PATIENT HAS A HIGH DISTRESS SCORE. Review remainder of report for potential causes of this distress and action the specific recommendations as detailed

**Emotional**	**Recommendation**
Wellbeing	Consider reasons for concern and, if required, refer to social work OR psychology OR clinical/health services for further assessment and care
Anxiety, Depression	Consider reasons for concern and compare to previous assessments (if available). If results are identical or increased, refer to social work OR psychology services for further assessment and care.

**Functional**	**Recommendation**
Lack of energy/tiredness, Staff acknowledgement of emotional needs	Clinically address as appropriate OR refer to relevant medical specialist OR allied health professional for further assessment and care
Transportation	Consider reasons for concern and, if required, refer to social work or centre-specific or community services for further assessment and care
Childcare, Housing, Insurance/Finance	Consider reasons for concern and, if required, refer to social work for further assessment and care

**Physical**	**Recommendation**
Appetite, Drowsy, Pain, Diarrhoea, Eating, Fatigue, Fevers, Feeling swollen	Clinically address as appropriate OR refer to relevant medical specialist OR allied health professional for further assessment and care

1. Distress (Distress Thermometer & Problem Checklist)

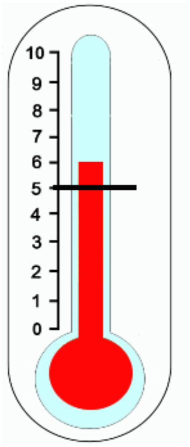

Practical problems:	Emotional problems:	Physical problems:	
Childcare	Depression	~~Appearance~~	~~Indigestion~~
Housing	Fears	~~Bathing/dressing~~	~~Memory/concentration~~
Insurance/financial	Nervousness	~~Breathing~~	~~Mouth sores~~
Transportation	Sadness	~~Changes in urination~~	~~Nausea~~
~~Work/School~~	Worry	~~Constipation~~	~~Nose dry/congested~~
~~Treatment decisions~~	Loss of interest in usual activities	Diarrhoea	~~Pain~~
**Family problems:**	~~Coping with illness~~	Eating	~~Sexual~~
~~Dealing with Children~~	~~Losing Hope~~	Fatigue	Skin dry/itchy
~~Dealing with partner~~	**Other:**	Feeling swollen	Sleep
~~Ability to have children~~	~~Spiritual/Religious~~	Fevers	Substance abuse
~~Family health issues~~		~~Getting around~~	Tingling in hands/feet

**** MOSAIQ eCERT LCTC *****

PROMPT Care
Patient Reported Outcome Measures for Personalised Treatment and Care

Patient Name: ZZZ, VICKY	MRN: 12345678	DOB: 27/06/2010	Date of Assessment: 11/12/2014

2. Symptoms (ESAS)

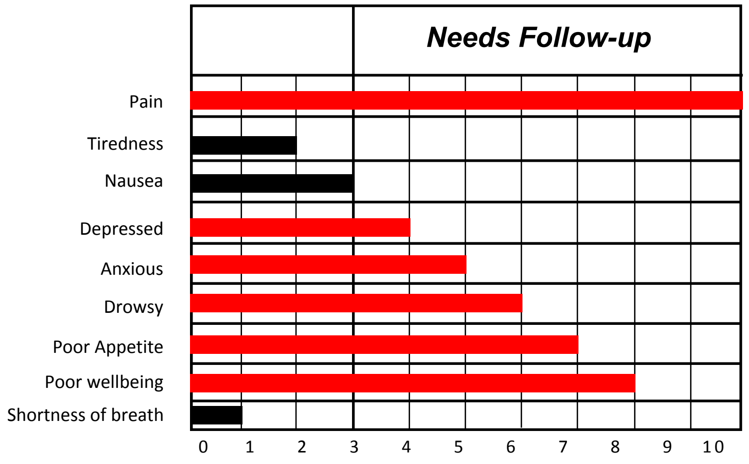

KEY

This issue is problematic for the patient

~~problem~~ Line through item = patient did not complete this item

4. Unmet Needs (SCNS-ST9)

Need	Issue
Psychological	Fears about the cancer spreading
	Uncertainty about the future
Health system and information	~~Being informed about your test results as soon as feasible~~
	Being informed about things you can do to help yourself to get well
Physical and daily living	Lack of energy/tiredness
	Not being able to do the things you used to do
Patient care and support	~~Reassurance by medical staff that the way you feel is normal~~
	Hospital staff acknowledging and show sensitivity to your feelings and emotional needs
Sexuality	~~Changes in sexual relationships~~
