# Supplementary material for: Development and Feasibility Testing of PROMPT-Care, an eHealth System for Collection and Use of Patient-Reported Outcome Measures for Personalized Treatment and Care: A Study Protocol
Source: JMIR Res Protoc. 2016 Nov 24;5(4):e227. doi: 10.2196/resprot.6459 (PMC5146324; doi:10.2196/resprot.6459)
Supplement: Multimedia Appendix 2 [file resprot_v5i4e227_app2.pdf]

**** MOSAIQ eCERT LCTC *****

PROMPT Care
Patient Reported Outcome Measures for Personalised Treatment and Care

Patient Name: ZZZ, VICKY | MRN: 12345678 | DOB: 27/06/2010

1. Distress (Distress Thermometer & Problem Checklist)

Date	DT score / 10
09/12/2014	8
11/12/2014	6

DT Score

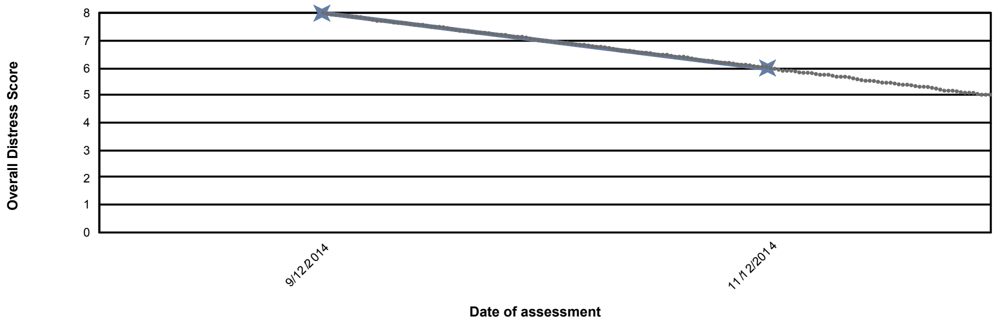

Date	Practical Problems						Family Problems				Emotional Problems								Other
	Child care	Housng	Insurance/Financial	Transportation	Work/School	Treatment Decision	Children	Partner	Having Children	Family Health	Depression	Fears	Nervousness	Sadness	Worry	Loss of interest in usual activity	Coping with Illness	Losing Hope	Spiritual/Religious concerns
09/12/2014				----	----	■	■	■	■	----	----			■	■		■		■
11/12/2014	■	■	■	■	----	----	----	----	----	----							----	----	■

Date	Physical Problems																					
	Appearance	Bathing / dressing	Breathing	Changes in urination	Constipation	Diarrhea	Eating	Fatigue	Feeling Swollen	Fevers	Getting around	Indigestion	Memory / concentration	Mouth sores	Nausea	Nose dry / congested	Pain	Sexual	Skin dry / itchy	Sleep	Substance abuse	Tingling in hands/feet
09/12/2014		■		■		■	----	----		■	■	■	----	----	----	----	----	----	----	----	----	----
11/12/2014	----	----	----	----	----	■	■	■	■	■	----	----	----	----	----	----	----	----				

**** MOSAIQ eCERT LCTC *****

PROMPT Care
Patient Reported Outcome Measures for Personalised Treatment and Care

Patient Name: ZZZ, VICKY	MRN: 12345678	DOB: 27/06/2010

2. Symptoms (Edmonton Symptom Assessment Scale)

0=Best; 10=Worst Possible									
Date	Pain /10	Tiredness /10	Nausea /10	Depressed /10	Anxious /10	Drowsy /10	Appetite /10	Wellbeing /10	Shortness of Breath /10
09/12/2014	7	4	3	8	----	7	8	6	3
11/12/2014	10	2	3	4	5	6	7	8	1

3. Unmet Needs (Supportive Care Needs Survey ST9)

No Need (1=N/A; 2= Satifisfied) | Some Need (3=Low Need; 4=Moderate Need; 5=High Need)									
	Psychological Need		*Health System & Information Need*		*Daily Living Need*		*Patient Care Need*		*Sexuality Need*
Date	Fears about the cancer spreading	Uncertainty about the future	Being informed about your test results as soon as feasible	Being informed about things you can do to help	Lack of energy / tiredness	Not being able to do the things you used to	Reassurance by medical staff that the way you feel is normal	Hospital staff acknowledging, and showing sensitivity to your feelings	Changes in sexual relationships
09/12/2014	4	5	1	2	----	----	1	2	4
11/12/2014	1	2	----	3	4	1	----	5	----
